# Enhancing Biosludge Dewaterability with Hemoglobin from Waste Blood as a Bioflocculant

**DOI:** 10.3390/polym12112755

**Published:** 2020-11-22

**Authors:** Hamed Ghazisaidi, Rafael A. Garcia, Honghi Tran, Runlin Yuan, D. Grant Allen

**Affiliations:** 1 Department of Chemical Engineering and Applied Chemistry, University of Toronto, Toronto, ON M5S 3E5, Canada; hamed.ghazisaidi@utoronto.ca (H.G.); honghi.tran@utoronto.ca (H.T.); runlin.yuan@mail.utoronto.ca (R.Y.); 2 United States Department of Agriculture, Agricultural Research Service, Eastern Regional Research Center, Biobased and Other Animal Coproducts Research Unit, 600 East Mermaid Lane, Wyndmoor, PA 19038, USA; rafael.garcia@usda.gov

**Keywords:** biosludge, dewatering, bioflocculant, hemoglobin, methylation

## Abstract

Synthetic polymers are widely used in the treatment of biosludge (waste activated sludge) to enhance its dewaterability. This paper discusses the results of a systematic study using hemoglobin (Hb) from animal blood and methylated hemoglobin (MeHb), a derivative in which a methyl group replaces the hydrogen carboxyl groups, to replace synthetic polymers to improve the dewatering efficiency of biosludge. With regular hemoglobin, no improvement in biosludge dewatering was found. With 10% of methylated hemoglobin per total solids content, however, the dry solids content of biosludge increased from 10.2 (±0.3) wt% to 15.0 (±1.0) wt%. Zeta potential measurements showed a decrease in the negative surface charge of the particles in biosludge from −34.3 (±3.2) mV to −19.0 (±2.1) mV after the treatment with methylated hemoglobin. This, along with an unchanged particle size distribution after conditioning, suggests that charge neutralization is likely the main cause of particle flocculation. With charges neutralized, the extracellular polymeric substances (EPS) around the biosludge flocs become loose, releasing the trapped water, thus increasing dewaterability.

## 1. Introduction

The pulp and paper industry uses a large amount of water in almost every step of its process, which creates various challenges such as wastewater discharge, treatment, and solids disposal [1,2,3,4,5]. The activated sludge process is a biological method often used in wastewater treatment plants (WWTPs) including pulp and paper mills, for secondary treatment [4]. This method utilizes aerobic microorganisms to break down organic substances into carbon dioxide, water, ammonia, etc. [1,4]. As a result of this process, large quantities of biosludge are generated, which have to be treated according to applicable regulations before final disposal [5]. In Canada, biosludge produced by the pulp and paper industry surpasses that produced by all municipal WWTPs combined [6,7,8], and its management accounts for as much as 60% of the pulp mill’s wastewater treatment cost [5,9].

The challenge in separating the water content is due to a variety of reasons such as the complex gel-like structure of biosludge held together with extracellular polymers (EPS), highly charged particles and others [10]. This three-dimensional biopolymer network can trap water molecules and cause the water content in biosludge to be above 98% [8,11,12], thus making it difficult to dewater [8,13]. Canadian pulp and paper mills alone produce over 1.5 million tons of dry solid sludge, and it is expected to grow significantly by 2020 [14]. Therefore, it is of economic and environmental interest to decrease the sludge volume so that post-treatment may be carried out more effectively [9,15].

Chemical conditioners are often used in pulp and paper mills to treat biosludge before mechanical dewatering to promote the release of water content including free and bound water [16,17]. Synthetic, water-soluble polymers are the most commonly used amongst all conditioners [18]; these same substances are used in other industries to flocculate colloidal suspensions. Although synthetic polymers dewater biosludge well with a low dosage, they are dose-sensitive and costly. Furthermore, they are derivative products of petroleum which means they are non-renewable material and can also be toxic to the ecosystem [18,19,20].

Bioflocculants represent a potential alternative to synthetic polymers in that they can be made from renewable resources and are generally non-toxic. Various bioflocculants have been tested for their flocculation activity but, in general, their performance is poor when used at dosages that are sufficient for synthetic polymers due to their low solubility, molecular structure, and low charge density [21,22,23]. Some examples of bioflocculants are starches, galactomannans, cellulose derivatives, gelatin, casein, soy protein, etc. [18,19,21,24]. Despite the numerous options available, an ideal bioflocculant needs to not only be effective in improving biosludge dewaterability, but also abundant in nature and easily recoverable.

Proteins show good potential to be used as bioflocculants for charged suspended particles and colloidal materials. The main flocculation mechanisms for proteins, similar to many other biopolymers, are interparticle bridging and electrostatic neutralization [25]. The net charge of a protein is affected by the pH of the medium surrounding the protein. When the pH value is above the isoelectric point, the protein is negatively charged. Conversely, when the pH value is below the isoelectric point, the net charge on the protein is positive [21].

Hemoglobin (Hb) is the most abundant blood protein. It is contained within the red blood cells and is easily isolated from other blood components [26,27]. In the US, approximately 2 million tons of animal blood are produced annually as a by-product of meat processing which is a rich source of protein (>90% on a dry basis) [28]. When Hb was tested on kaolin clay or lignin suspensions, it showed promising flocculation with a relatively low dosage [29]. Hb modified by methylation of its carboxylic acid groups showed greatly improved flocculation of kaolin suspensions, and functioned over a wider pH range [30,31]. Since biosludge particles are negatively charged, like kaolin and lignin, Hb and MeHb may also be effective in flocculating biosludge and promoting dewatering.

The aim of this study was to explore the possibility of using Hb as a conditioner to improve dewaterability of biosludge from pulp and paper mills and to investigate the effect of methylation on the performance of hemoglobin as conditioner. In addition, main properties of biosludge such as particle size distribution, surface charge, EPS content and bound water content was evaluated to understand how Hb and MeHb affect biosludge particles and floc structures.

Since charge neutralization is considered one of the most important flocculating mechanisms [32], Hb is methylated to further enhance charge neutralization [30]. Both regular and methylated hemoglobin (MeHb) were tested at different dosages with measuring dry solids content in the cake, zeta potential, particle size, EPS concentration, and the amount of bound water at each stage of the experiment to provide insight into the mechanism of flocculation.

## 2. Materials and Methods

### 2.1. Biosludge

A biosludge sample was obtained from the secondary clarifier of a Canadian pulp and paper mill, which produces pulp, paper and advanced products utilizing sulfite pulping and mechanical pulping. The biosludge sample was collected and then stored in a cold room at 4 °C prior to use. Prior to experiments, the biosludge was well mixed to ensure sample homogeneity and a desired amount was taken out to reach room temperature before conditioning. The total solids content (TS) of the raw sludge was measured to be 15.1 ± 0.2 g/L and all experiments were conducted using the same batch of biosludge.

### 2.2. Crown Press

**Figure 1:**
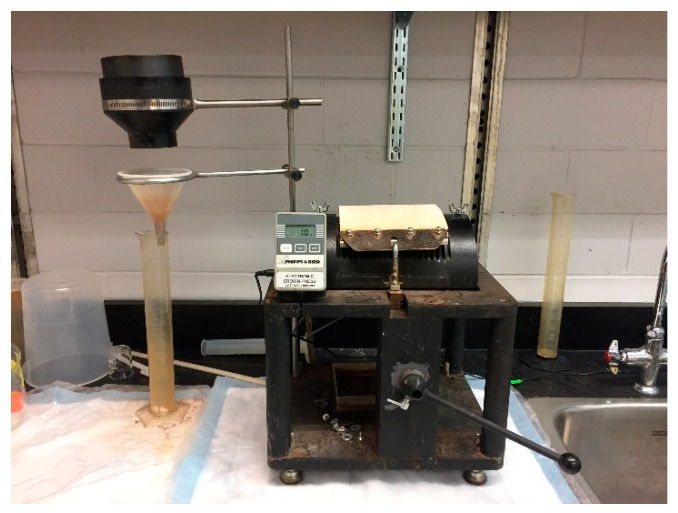
Crown Press—A bench-scale press to assess the dewaterability of biosludge.

A bench-scale crown press (Phipps & Bird Inc., Richmond, VA, USA) was used to simulate belt filter presses used in industry for sludge dewatering (Figure 1). This equipment has two stages for separation: gravity thickening followed by belt pressing. In the gravity thickening step, a double-layered woven fabric was used as the filter placed on top of a filter holder. Biosludge was poured onto the filter and the filtrate was collected in a graduated cylinder. The wet residue from the gravity filtration was collected for mechanical compression where it was placed in between two press filters, sequentially increasing applied force on the residue for 10 s at 80 lb, 120 lb, and 150 lb. The pressed residue or cake was weighed before and after drying in the oven to determine its total solid content (TS) and infer the performance of the conditioner.

### 2.3. Methylation of Hemoglobin

Methylated hemoglobin was prepared using the method developed by Fraenkel-Conrat and Olcott [30]. In summary, 3% (w/v) lyophilized bovine hemoglobin (Sigma-Aldrich, St. Louis, MO, USA) was suspended in methanol (Sigma-Aldrich, St. Louis, MO, USA) followed by the addition of hydrochloric acid (Sigma-Aldrich, St. Louis, MO, USA) with the final concentration being 0.8 mol/L. The mixture was then shaken continuously for 48 h at room temperature. Afterwards, the mixture was centrifuged at 10,000× *g* for 15 min and was washed twice with methanol and resuspended in water. Subsequently, the suspension was dialyzed through 6000–8000 molecular weight cut off membrane against dilute hydrochloric acid to recover the methylated bovine hemoglobin (MeHb).

### 2.4. Zeta Potential

Zeta potential of pure and conditioned biosludge was measured using Zeta Compact Z9000 (CAD Instruments, Les Essarts le roi, France) to compare the change in surface charge of the particles when subjected to different proteins and protein dosages. A ten-fold dilution with water was performed prior to the measurement to ensure the visibility of the particles.

### 2.5. Particle Size Analysis

A laser diffraction-based instrument (Mastersizer S, Malvern Panalytical Ltd., Malvern, UK) was used to measure the size distribution of particles and flocs. It is equipped with a 300 mm lens that makes it possible to measure the size of particles with a range of 0.05 to 900 μm. Raw and conditioned sludge samples were analyzed during and after conditioning and obscuration of 30% (±1) was used for all samples.

### 2.6. EPS Stratification

Flocs in biosludge consist of microbial communities mainly surrounded by EPS. EPS can be classified into three groups: tightly bound (TB), loosely bound (LB), and soluble. Biosludge was first stratified to extract and separate EPS into soluble EPS, loosely bound EPS (LB-EPS), and tightly bound EPS (TB-EPS). The stratification method used was the one described by Guang et al. [33] with a slight modification. First, a buffer solution containing Na_3_PO_4_, NaH_2_PO_4_, NaCl, and KCl (Sigma-Aldrich, St. Louis, MO, USA) at a molar ratio of the salts of 2:4:9:1was generated using deionized water and the pH was adjusted to 7. The conductivity of the solution was adjusted with water until it matched that of the biosludge. The sludge was first centrifuged at 2000× *g* at 4 °C for 15 min, the supernatant was collected as the soluble EPS, while the sediment was resuspended to its original volume using the buffer solution. The resuspended solution was centrifuged at 5000× *g* for 15 min, the supernatant was collected as LB-EPS, and the sediment was resuspended again with the buffer solution to its original volume. The resuspended sediment was subjected to ultrasound treatment for 20 min (using Qsonica Q700, Qsonica, Newtown, CT, USA) with 13 mm probes set at 85 amplitude. The ultrasound-treated samples were then centrifuged at 20,000× *g* for 20 min. The supernatant was collected as TB-EPS, and the remaining solids were disposed. All EPS samples were filtered using a 0.2 µm syringe filter before EPS assay.

#### 2.6.1. EPS Protein Content Measurement

In order to measure the protein content of the EPS, stratified EPS samples were analyzed using the modified Lowry method [34]. In brief, three solutions were prepared prior to the measurement. Solution A contains 0.2 g of potassium sodium tartrate and 10 g of sodium carbonate dissolved in 50 mL of 1 N sodium hydroxide, then diluted to 100 mL with water. Solution B contains 2 g of potassium sodium tartrate and 1 g of copper (II) sulfate pentahydrate dissolved in 90 mL water and 10 mL 1 N sodium hydroxide. Solution C contains 1 mL of Folin-Ciocalteau pheno reagent and 14 mL water. After solution preparation, a 96-well plate was used for absorbance measurement. For each stratified EPS sample, 40 µL of the sample, 36 µL of solution A, 4 µL of solution B, and 120 µL of solution C were added into each well. The same procedure was repeated but without the addition of solution B. After adding all the components and pipetting up and down for mixing, the samples were placed in a 50 °C water bath for 10 min. After that, the samples were cooled down to room temperature before absorbance reading (using SpectraMax M2, Molecular Devices Corporation, San Jose, CA, USA) at 650 nm. The samples with solution B added measured $A_{total}$, the one without it measured $A_{blind}$. The protein absorbance was calculated as:

(1) $$A_{Protein}=1.25\left(A_{total}-A_{blind}\right).\;$$

For the calibration curve, the above steps were repeated with the EPS sample replaced by Bovine Serum Albumin (BSA, Molecular Biology Grade, Thermo Fisher Scientific, Waltham, MA, USA) with known concentration.

#### 2.6.2. EPS Carbohydrates Measurement

Total and soluble carbohydrates in samples were measured using the Anthrone method [35]. The anthrone solution was prepared by adding 0.1 g of anthrone reagent (Sigma-Aldrich, St. Louis, MO, USA) into 50 mL of concentrated sulfuric acid (Sigma-Aldrich, St. Louis, MO, USA). Then, 150 µL of anthrone solution and 50 µL of sample was added to a 96-well plate followed by incubation at 4 °C for 10 min, then at 103 °C for 20 min. Absorbance was read at 620 nm at room temperature. The calibration curve was prepared by replacing the sample with glucose. All steps were performed in a dark room, since anthrone solution is very sensitive to light.

### 2.7. Bound Water Content

The distribution of water in the biosludge can help describe the extent to which a sample of biosludge is amenable to further dewatering. The typical classification is to consider the water as being either free or bound water. Free water is not attached to sludge solids and is relatively easy to remove by mechanical methods while the bound water is not and undergoes phase change at a different temperature [11]. The bound water content of the biosludge conditioned with different doses of MeHb was measured by differential scanning calorimetry (DSC) 250 equipment (Discovery DSC 250, TA Instruments, New Castle, DE, USA). DSC was used to measure the enthalpy of freezing the free water content of the sludge sample by cooling it from 25 °C to −60 °C. The rate of cooling was 5 °C/min and a peak in the thermograph appeared around −7 °C which was due to freezing the free water [36]. After the sample reached −60 °C, it was heated up back to 25 °C. The amount of water can be obtained by using the equation below:

(2) WB=WT−ΔHΔH0. 

where $W_{B}$ and $W_{T}$ are the bound and total water content of the sludge samples, respectively. ΔH is the amount of enthalpy change due to freezing the free water in the biosludge samples, and $\Delta H_{0}$ is the standard enthalpy for melting of ice (334.7 J/g).

## 3. Results

### 3.1. Effect of Hemoglobin and Methylated Hemoglobin on Cake Dry Solids Content

**Figure 2:**
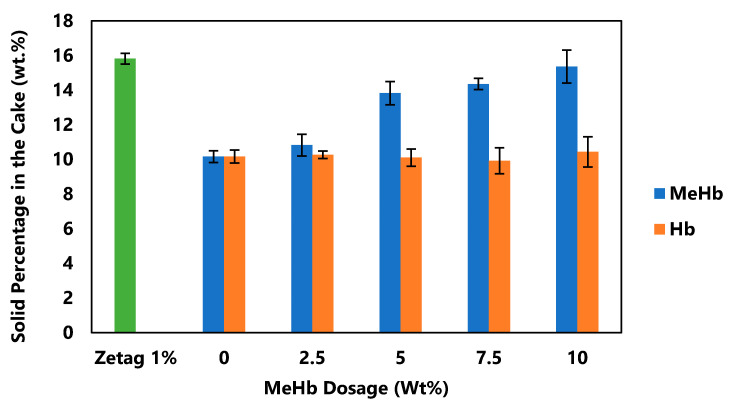
Effect of hemoglobin and methylated hemoglobin on cake dry solids content at different dosages.

After drying biosludge in an oven at 104 °C for 24 h, the total solids (TS) was measured by measuring the weight difference between wet and dry sludge. Dosage of the bioflocculants was defined to be the dry weight percent of conditioners to sludge TS and was applied from 0 to 10%. After conditioning the biosludge with both hemoglobin and methylated hemoglobin, the dry solids content in the cake after the crown press was measured, as shown in Figure 2.

The addition of methylated hemoglobin steadily increased the dry solids content from 10.2 (±0.3) wt% to 15.3 (±1.0) wt%. On the other hand, the addition of hemoglobin did not show any significant improvement (*p* > 0.05) with the dry solids content fluctuating around 10 wt%. All the results in this study was analyzed statistically by Minitab Software using ANOVA method.

The inability of hemoglobin to flocculate was previously suggested by Essandoh et al. [30] on other types of suspensions such as kaolin and lignin suspension. They showed that at a pH above 5.5, the ability of hemoglobin to perform as a flocculant drops rapidly [30]. Since the isoelectric points of Hb and methylated Hb are 6 and 8, respectively [29], it is expected that lowering pH would lead to a higher positive charge on proteins and therefore a better flocculation performance towards negatively charged particles. The pH value of sludge in this study was measured to be 6.9 which was higher than Hb isoelectric point. Hence, the inability of hemoglobin to flocculate biosludge was expected as further confirmed by the results for TS of the cake.

The major difference between Hb and MeHb is the replacement of the carboxylic acid groups with methyl ester groups [37]. This replacement increases the net positive charge of the hemoglobin molecule, resulting in better flocculation, charge neutralization of solid particles, and improved dewaterability. As it is shown in Figure 2, by adding 10% of MeHb, it was possible to achieve almost the same dry solids content as 1% of Zetag8165 which is a strong common synthetic polymer for biosludge treatment. Although MeHb is able to provide the same results for dewaterability as the commercial Zetag8165, it requires a much higher dose and so is not currently cost competitive.

### 3.2. Effect of Methylated Hemoglobin on Particle Size

**Figure 3:**
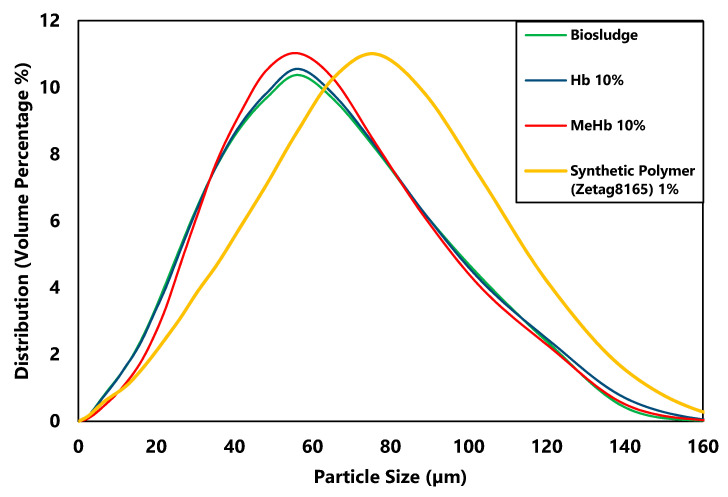
Particle size distribution of raw biosludge, conditioned biosludge with 10% of hemoglobin and methylated hemoglobin.

**Figure 4:**
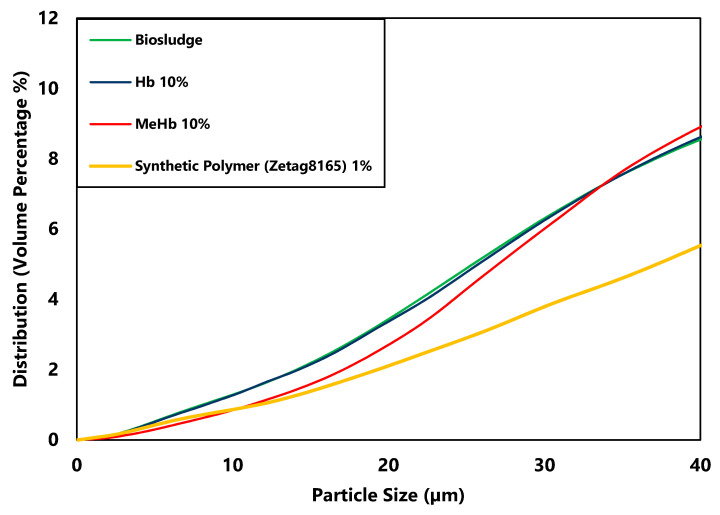
Particle size distribution of raw biosludge and conditioned biosludge with 10% of hemoglobin and methylated hemoglobin within the range of 0–40 µm.

Agglomeration can be observed by measuring the particle size of flocs in biosludge after conditioning as is shown in Figure 3 and Figure 4.

Figure 3 shows the difference between biosludge and conditioned biosludge at 10% dosage of Hb and MeHb. The most abundant size present in this biosludge was around 55 µm, and the largest was 160 µm. Although the upper and lower limits remained the same after conditioning, with MeHb, there was a slight shift from small particles (<30 µm) to medium size particles (30–70 µm). On the other hand, for Hb, no change in the particle size distribution was observed, which is consistent with the results from crown press.

Using MeHb resulted in a statistically significant (*p* < 0.05) change in the particle size, especially for smaller particles to average size particles. However, comparing to the particle size change by using Zetag8165, this shift appears to be small and does not explain the change in the dry solids content and hence the improvement in the dewaterability. It seems that instead of the aggregation of the bigger particles, methylated hemoglobin may cause smaller particles in the size range of 0–40 µm (shown in Figure 4) to adhere to the larger particles, which explains the small change in particle size.

### 3.3. Effect of Protein Dosage on Zeta Potential

**Figure 5:**
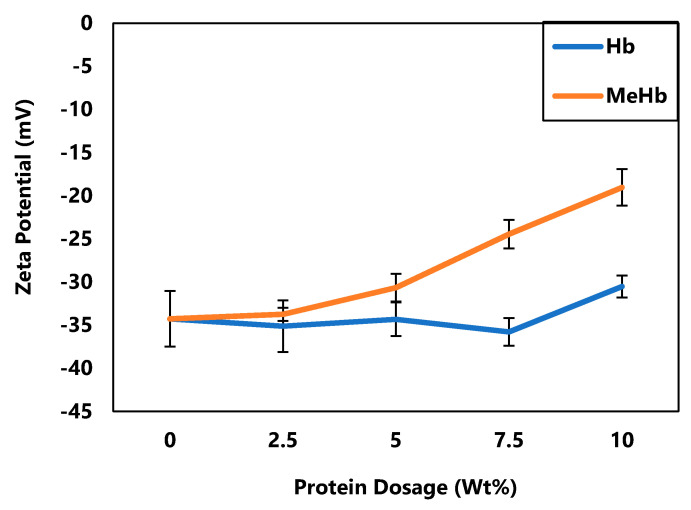
Zeta potential measurement of conditioned sludge with hemoglobin and methylated hemoglobin.

Figure 5 shows the Zeta potential of biosludge conditioned with both regular and methylated hemoglobin from 0% to 10%. There was a significant decrease in negative surface charge for MeHb (*p* < 0.05) while no significant change was observed for Hb (*p* > 0.05). Since no improvement in dry solids content was observed when conditioned with hemoglobin, change in zeta potential was also not expected, except when a dosage of 10 wt% hemoglobin was used. On the other hand, methylated hemoglobin showed consistent change on zeta potential from −34.3 (±3.2) mV to −19.0 (±2.1) mV with increasing dosage.

This decrease in zeta potential of biosludge when conditioned with methylated hemoglobin is consistent with the increase in dry solids content, suggesting that there is a direct correlation between surface charge and biosludge dewaterability. The higher zeta potential for methylated hemoglobin likely resulted from the process of methylation. During methylation, some of the carboxylic acid groups in hemoglobin are replaced by methyl ester groups, which results in an increase in basicity and net positive charge of the hemoglobin molecule, hence, methylated hemoglobin promotes more charge neutralization towards negatively charged particles in the biosludge [30].

### 3.4. Effect of MeHb on Extracellular Polymeric Substances (EPS)

**Figure 6:**
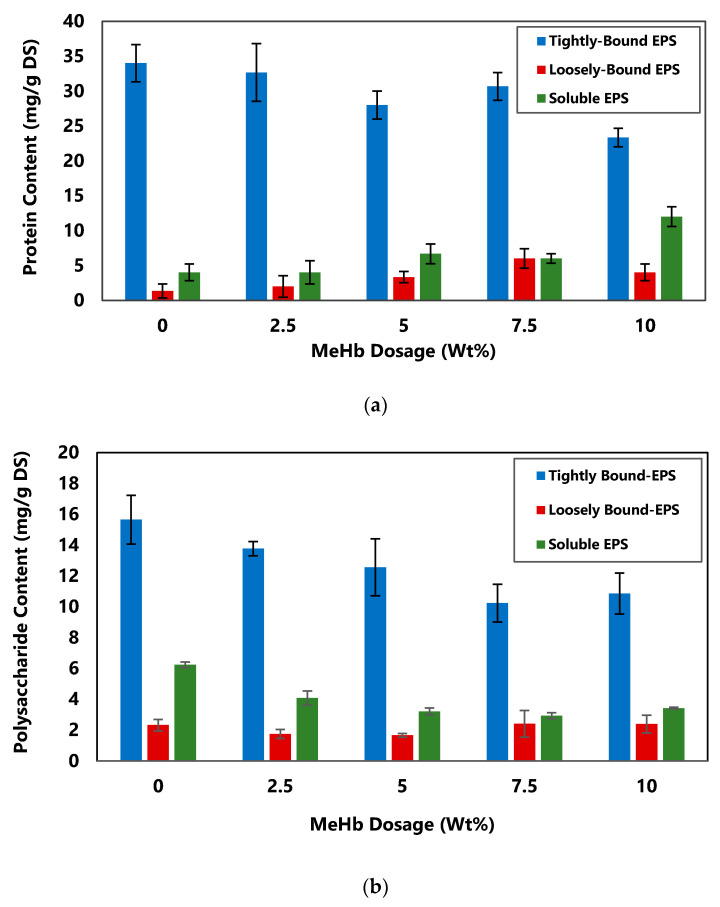
(**a**) Protein content and (**b**) polysaccharide content of EPS in biosludge at different dosage of methylated hemoglobin.

As shown in Figure 6, the composition of the EPS changed significantly (*p* < 0.05) by conditioning with MeHb. Microorganisms in untreated biosludge were mainly surrounded by TB-EPS which had a downward trend after adding MeHb. Raw biosludge had a concentration of 15.6 mg/g DS and 34 mg/g DS of polysaccharides (PS) and proteins (PN) in the TB-EPS layer, respectively. After MeHb conditioning, the distribution of PS and PN in TB-EPS decreased to 10.8 mg/g DS and 23.3 mg/g DS respectively as the dosage increased while the composition of LB-EPS did not change significantly (*p* > 0.05). However, for PN in the soluble EPS, an upward change was observed which is likely due to having a portion of MeHb in the soluble fraction. MeHb is a positively charged small molecule comparing to the solid particles in the sludge, therefore most of its molecules may be trapped and end up in the solid residue after the centrifugation. However, it is likely that some of the MeHb which are bonded to the outer layer of the flocs end up in the extract for soluble EPS. On the other hand, for PS, the same declining trend was observed as the dosage of MeHb increased.

It has been reported that the dewaterability of sludge is significantly affected by PN and not PS. However, having less PS especially in the soluble fraction can improve the dewaterability [38]. Similar to the results for the dry solids content, the trends for both PN and PS in all different layers of EPS suggests that by increasing the dosage of MeHb up to 10%, a plateau was not reached. Therefore, it is possible that by increasing the dosage furthermore, the change in PS and PN as well as the dry solids content continue.

With the solubilization of EPS, it is likely that the 3-dimension structure of the flocs changed and the flocs became looser and more open. TB-EPS was released into the bulk solution resulting in a smaller repulsive force between the flocs leading to a better settlement, which improves biosludge dewaterability.

### 3.5. Change in Bound Water Content

**Figure 7:**
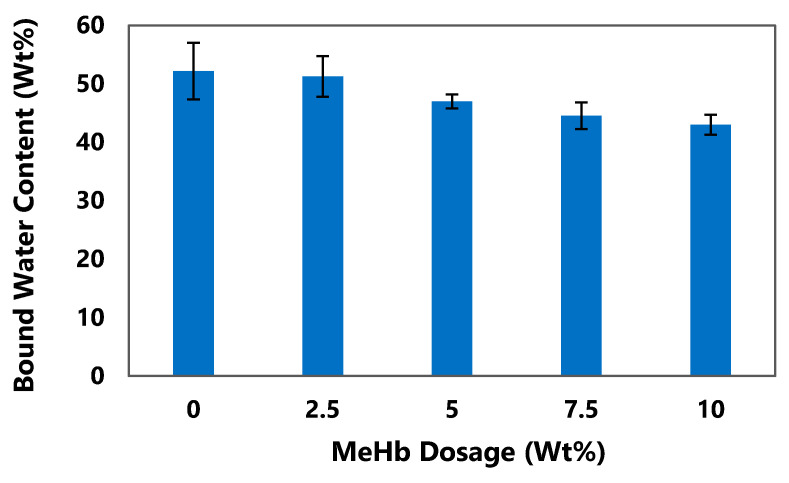
Bound water content in biosludge at different dosage of methylated hemoglobin.

Figure 7 shows the measured bound water percentage at different MeHb dosages. The bound water content decreased significantly (*p* < 0.05) with an increase in MeHb dosage. Pure biosludge has a bound water content of 52% which decreased consistently as MeHb dosage increased. At 10% dosage, the bound water content was reduced to 43%. The change in the bound water content was possibly due to the solubilization of EPS. As TB-EPS was disrupted and the structure of the flocs was changed, the ability of the flocs to retain water decreased, causing the bound water to release into the bulk solution. The results in Figure 7 suggests that by using MeHb it was possible to separate a portion of the bound water content which is the most difficult type of water in the sludge to remove and therefore improve the biosludge dewaterability.

## 4. Conclusions

In this work, we demonstrated that methylated hemoglobin improves biosludge dewaterability, as shown by the constant increase in dry solids content with increasing dosage. In addition, since hemoglobin showed no improvement in dewaterability, we conclude that the methylation process is key to promoting the flocculation potential of hemoglobin towards biosludge particles.

Although the particle size did not change significantly, the negative surface charge of the particles after conditioning decreased consistently suggesting the flocculation mechanism is charge neutralization. It is likely that by changing the surface charge, the EPS structure was disrupted and started to loosen, releasing the trapped water into the bulk solution. The bound water content measurement showed that at 10% dosage, almost 10% of the bound water was converted to free water, improving the dewaterability of biosludge.

In conclusion, methylated hemoglobin performs as flocculant through charge neutralization. However, the high dosage required to achieve a desirable dewaterability may not be economically feasible. Further optimization will be carried out to improve this sustainable approach by varying other important factors such as pH, mixing with primary sludge and using a dual conditioning system.
